# A Comparative Histopathology, Serology and Molecular Study, on Experimental Ocular Toxocariasis by *Toxocara cati* in Mongolian Gerbils and Wistar Rats

**DOI:** 10.1155/2013/109580

**Published:** 2013-08-29

**Authors:** Mohammad Zibaei, Seyed Mahmoud Sadjjadi, Mehdi Karamian, Shoji Uga, Ahmad Oryan, Seyed Hamidreza Jahadi-Hosseini

**Affiliations:** ^1^Department of Parasitology and Mycology, School of Medicine, Alborz University of Medical Sciences, P.O. Box 31485561, Karaj, Iran; ^2^Department of Parasitology and Mycology, School of Medicine, Shiraz University of Medical Sciences, P.O. Box 713451735, Shiraz, Iran; ^3^Basic Sciences in Infectious Diseases Research Center, School of Medicine, Shiraz University of Medical Sciences, P.O. Box 713451735, Shiraz, Iran; ^4^Department of Microbiology, School of Medicine, Birjand University of Medical Sciences, P.O. Box 917751365, Birjand, Iran; ^5^Department of Parasitology, Faculty of Health Sciences, Kobe University Graduate School of Health Sciences, P.O. Box 6540142, Kobe, Japan; ^6^Department of Pathology, School of Veterinary Medicine, Shiraz University, P.O. Box 713451735, Shiraz, Iran; ^7^Department of Ophthalmology, Khalili Hospital, Shiraz University of Medical Sciences, P.O. Box 713451735, Shiraz, Iran

## Abstract

The aim of this study was to compare the performance of three in-house diagnostic tests, that is, histopathology, enzyme-linked immunosorbent assay (ELISA), and polymerase chain reaction (PCR), for the diagnosis after experimental infection with *Toxocara cati*. Twenty Mongolian gerbils and Wistar rats were divided into ten groups (*n* = 2/group). *Toxocara cati* infections were established in Mongolian gerbils and Wistar rats by administering doses of 240 and 2500 embryonated *Toxocara cati* eggs by gavage, respectively. Tissue sections were stained with Haematoxylin and Eosin and observed under the light microscope. Sera and vitreous fluid collected from separate infected groups were tested against *Toxocara cati* antigens, for 92 days postinfection. Genomic DNA was extracted from formalin-fixed paraffin-embedded (FFPE) blocks, and aqueous fluids belong to the animals. The histopathology test gave negative results among the groups of animals examined between 5 and 92 days postinfection. The ELISA results showed that anti-*Toxocara* antibodies have risen between 7 and 61 days postinfection in sera and vitreous fluid in the animals infected, respectively. Analysis of PCR products revealed positive band (660 bp) in the orbital tissue infected Mongolian gerbils at 5 days postinfection. Of the three evaluated methods, the PCR could be recommended for scientific and laboratory diagnoses of toxocariasis in experimentally infected animals.

## 1. Introduction

Toxocariasis is one of the most commonly reported zoonotic helminthic infections in the world. Both *Toxocara canis* (a round worm of dogs) and *Toxocara cati* (common round worm of cats) cause toxocariasis. Ingestion of *Toxocara* species eggs containing third-stage larvae, due to geophagia, pica, and the consumption of contaminated raw meat or liver, is considered as possible cause of visceral larva migrans (VLM) or ocular larva migrans (OLM) [1, 2].

Ocular infection with *Toxocara* larvae species is uncommon but of interest to ophthalmologists because it largely affects the young people in whom it can cause significant ocular morbidity or even blindness. The clinical sign of ocular toxocariasis in human often includes diminished vision, leukocoria, red eye, and strabismus. The diagnosis of suspected ocular toxocariasis is supported by the presence of chorioretinal or focal lesions in posterior eye segment in the presence of positive serology [3, 4]. *Toxocara cati (T. cati) *is the common worm of cats and its third-stage larvae are possible cause of OLM. Several studies have been reported that human can be infected by the ingestion of *T. cati* embryonated eggs with contaminated soil or eating larvae within paratenic hosts including birds and small rodents. There is little finding on the location of *T. cati* larvae in paratenic hosts including the eye as causative agents of human toxocariasis [5, 6].

In the last few years, several authors have approached diagnosis through the specific detection of antigens or antibodies [7, 8]. Nevertheless, the histopathological demonstration of larvae is relatively intensive, especially due to low larvae burden [9, 10]. Thus, the development of new and more sensitive diagnostic tools is needed. Desirably, theis new approach should permit the diagnosis in early infection, to prevent the development of severe pathologies associated with later infection. Application of molecular methods for the diagnosis of *Toxocara* infections has been increasing. Several studies have reported the usefulness of polymerase chain reaction (PCR) for the characterization and the diagnosis of toxocariasis compared to other parasitic diseases in natural or experimentally infected animals [11, 12]. 

Based on the widespread use of polymerase chain reaction for the diagnosis of parasitic diseases, and to the best of our knowledge that there are no reports about of use of PCR in diagnosis of ocular toxocariasis, this study was designed to evaluate PCR for being used in the diagnosis after experimental ocular infection with *T. cati *third-stage larvae, compared to histopathology and Enzyme-Linked Immunosorbent Assay (ELISA).

## 2. Materials and Methods

### 2.1. Animals and Experimental Infections

Ten Mongolian gerbils (one-month-old males) and ten Wistar rats (one-month-old males) were assigned in ten experimental groups (*n* = 2/group): each two animals of the same, were infected orally with approximately 240 and 2500 embryonated *T. cati* eggs, respectively [6, 13–15]. The eggs were derived from adult female worms and were embryonated according to our previous paper [16]. The animals were maintained under standard conditions, in an environment with controlled temperature and humidity. The inoculated animals were euthanized on days 5, 30, 49, 70, and 92 postinfection (PI). The vitreous fluid which is a jelly compound was taken from the eyeballs of infected animals, using a 22-gauge needle linked to a powerful syringe and stored at −30°C until used. The experimental protocol was approved by the Ethical Committee of the Shiraz University of Medical Sciences.

### 2.2. Preparation of *T. cati* Excretory-Secretory (ES) Antigens

ES antigens for ELISA assay were obtained from third-stage larvae (ES/L_3_). Adult worms of *T. cati* were collected from the intestines of infected stray cats and the eggs were isolated from uteri of female worms, were decoated by sodium hypochlorite (7% w/v), and were embryonated in 2.5% formalin/ringer solution while being incubated at an atmosphere of 5% CO_2_ in 25°C for 3 weeks [16]. The larvae were collected aseptically from a Baermanns apparatus. ES antigens of *T. cati* were prepared from third-stage larvae by the method of de Savigny (1975) with modification [17].

### 2.3. Pathological Study

Samples of eyeball from the infected animals were fixed in 10% neutral buffered formalin, dehydrated with graded ethanol, and embedded in paraffin. Tissue sections of 5 *μ*m thickness were stained with Haematoxylin and Eosin (H & E) and after mounting, were observed under the light microscope.

### 2.4. Immunological Study

ELISA was carried out in flat-bottom96 microplates. The ES antigen was collected from culture supernatant of *T. cati* third-stage larvae as described. The plates were coated with 100 *μ*L of ES antigen in coating buffer (0.05 M carbonate bicarbonate-buffer, pH 9.6) and incubated at 4°C overnight. Excess antigen was removed by washing the plate three times in phosphate buffered saline-Tween 20 (PBST, pH 7.4 containing 0.05% Tween 20). Excess binding sites were blocked for 1 hr at 37°C with 100 *μ*L of bovine serum albumin (1% dilution in PBS). The wells were washed, and then 100 *μ*L of diluted serum (1 : 100 in PBS) and vitreous humor (1 : 2 in PBS) were added to each well and incubated for 1 hr at 37°C. The plates were washed as before, and 100 *μ*L of horseradish peroxidase-conjugated sheep anti-mouse IgG at a 1/1000 dilution in PBST was added and incubated for one hour at 37°C. After washing, the plates were incubated with chromogen/substrate (100 *μ*L/well ABTS peroxidase substrate) and the reaction was terminated with peroxidase stop solution (50 *μ*L, diluted 1 : 5) after 5 min. The absorbance at 405 nm was monitored with a MUNK microplate reader. The cutoff point was set as 2SD above the mean of control samples.

### 2.5. DNA Isolation and PCR Analyses

Extraction of DNA was done from formalin-fixed paraffin-embedded (FFPE) blocks, and aqueous fluids belong to gerbils and rats. FFPE blocks were cut and deparaffinized using Xylol due to standard protocols. Genomic DNA was isolated from individual samples using QIAamp DNA Mini and Blood Mini Handbook Kit (Qiagen, Hilden, Germany) according to the manufacturer's instruction. The specific forward primer JW4 (5′-ACTGTCGAGGATGAGCGTGA-3′) [18] was used with NC2 primer (reverse: 5′-TTAGTTTCTTTTCCTCCGCT-3′) [11] to amplify partial ITS-1, complete 5.8S, and ITS-2 rDNA of *T. cati*. PCR reaction was performed in 10 mM Tris-HCL, pH 8.8, 1.5 mM MgCl_2_, 50 mM KCl, 0.1% Triton X-100, dNTP 200 *μ*M, primers 1 *μ*M, and 1U Taq polymerase per 25 *μ*L. The reaction was carried out under the following conditions: 94°C for 45 s, 58°C for 45 s, 72°C for 90s, and final extension 72°C for 10 min. Corresponding PCR products were electrophoresed in 1.4% agarose gel with 0.5 *μ*g/mL ethidium bromide, and a 100-bp ladder was used as DNA sizemarker for estimating the size of the amplicons and photographed using a gel documentation system (UV Transilluminator).

## 3. Results

In an attempt to identify the optimal procedure for the diagnosis of ocular *Toxocara* infection, the performance and agreement among the three different methods, namely, histopathology, ELISA, and PCR methods, were evaluated.

### 3.1. Pathological Observation

**Figure 1:**
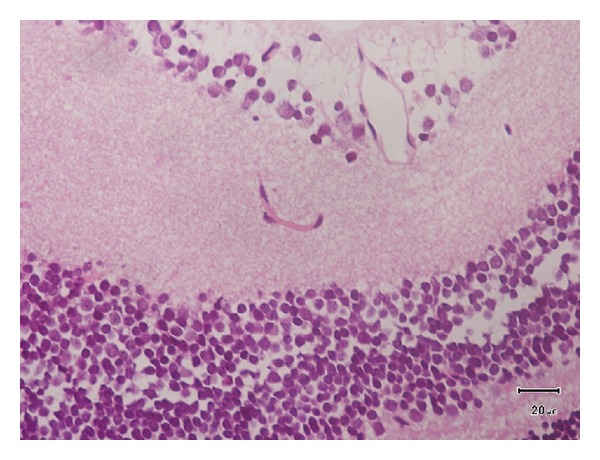
The negative reaction in the retina of the infected animals (paraffin section stained, H with E).

Figure 1 corresponds to image of the ocular structure of an infected animal. In none of the studied animals, we observed peripheral granuloma in the posterior pole or retrolental hyaline membranes. 

### 3.2. ELISA for Anti-*Toxocara* Antibodies

**Figure 2:**
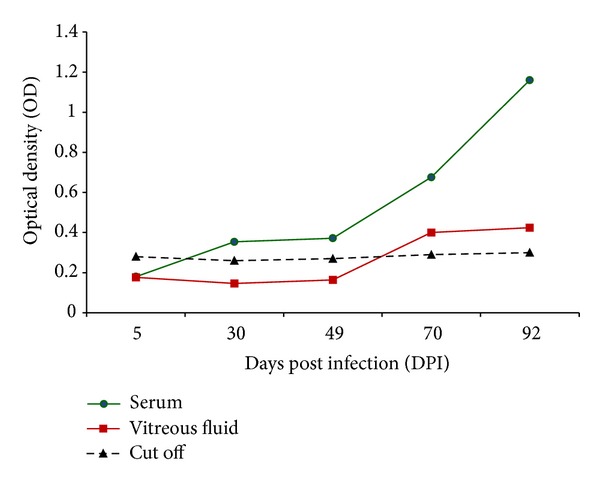
Development of specific *Toxocara cati* L2S ES antigens in infected Mongolian gerbils and Wistar rats with 240 and 2500 *T. cati* embryonated eggs, respectively. OD values of uninfected animals were lower than the negative cutoff 0.260, and those of infected animals were higher than positive cutoff 0.300.

The ELISA results were positive in sera and aqueous fluid for Mongolian gerbils and Wistar rats examined at 1 and 8 weeks PI, respectively. Specimens with an OD (optical density) reading greater than 0.300 and less than 0.260 can be interpreted with assurance as positive and negative, respectively. Caution may be necessary with specimens with inter with mediate values. At the seventh day PI, the test was positive in 15 out of 20 animals (75% sensitivity), where the animals showed anti-*Toxocara* antibodies in sera. The test was positive at an OD greater than 0.310 and higher in 10 Mongolian gerbils and 5 Wistar rats. At the sixty-one day PI the test was positive for aqueous fluid in 11 out of 20 animals (55% sensitivity), where the test was positive at an OD of 0.300 or more in nine Mongolian gerbils and two Wistar rats (Figure 2).

### 3.3. PCR Analysis

**Figure 3:**
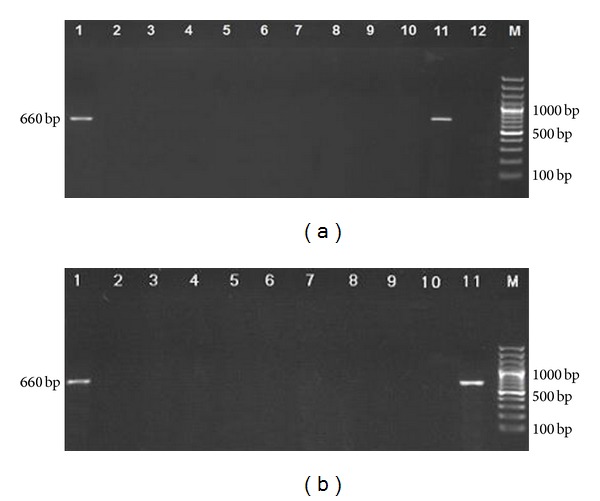
(a) Analysis of PCR products (eye tissue of infected animals) by 1.4% agarose gel electrophoresis. Lanes 1–5: days 5 to 92 PI of Mongolian gerbils; lanes 6–10: days 5 to 92 PI of Wistar rats; lane 11: DNA of *Toxocara cati* adult worm as positive control; lane 12: negative control; lane M: 100–1500 bp DNA size marker. (b) DNA amplification of vitreous fluid samples of infected animals by PCR on 1.4% agarose gel. Lanes 1–5: days 5 to 92 PI of Mongolian gerbils; lanes 6–10: days 5 to 92 PI of Wistar rats; lane 11: DNA of *Toxocara cati* adult worm as positive control; lane M: 100–1500 bp DNA size marker.

Analysis of PCR products revealed positive band (660 bp) in the orbital tissue infected Mongolian gerbils at 5 days PI. The DNA of *T. cati* was found in the vitreous fluid of experimentally infected Mongolian gerbils. No positive band was shown in samples of Wistar rats (Figure 3). 

## 4. Discussion

Toxocariasis is an important infection among socioeconomically disadvantaged young children and is low in adults. Ocular larva migrans occurs when *Toxocara* larvae reach eye tissue, where they induce inflammatory reaction, frequently without the sign or symptoms that accompany the visceral toxocariasis [19–22]. The diagnosis of ocular larva migrans is based on retinoscopic and retinographic studies, and is confirmed by the finding of larvae in lesions [23]. The presence of anti-*Toxocara* antibodies or antigens in vitreous humor has been reported to be a sensitive method for detecting toxocariasis in infections or human ocular toxocariasis [25].

In this study we compared the performance of three different diagnostic techniques including: histopathology, indirect ELISA and PCR on eyeball and retina tissue during the infection of Mongolian gerbils and Wistar rats from 5 to 92 days PI.

The infection with *T. cati *in the animals not caused important histological alternative in cellular structures of the eye. Histopathologic examination did not detect foreign bodies or tumors in the affected region, and the ophthalmic examination did not suggest an infectious process. Serological testing is the primary diagnostic tool in patient suspected for ocular and visceral larva migrans, but these tests are insufficient, as cross-reactions may occuring. For example, cross-reactivity has been reported between *T. canis* and *Trichinella spiralis*, and *T. canis* and *Ascaris lumbricoides* [26]. Sommerfelt et al. (2006) detected specific antibodies against *T. canis* with ELISA, in eighteen pigs at 7 days PI, with high titers persisting over an 84-day period [27]. In current study, an immunological response in sera was early, 7 days PI, and was maintained throughout the study (92 days) and these results are similar to those of other authors. Beside, from 61 days of the infection antibodies in vitreous fluid were detected. 

An alternative approach to detection of toxocariasis is to use a molecular-based-method such as PCR. When PCR was applied to eye tissue and aqueous fluid of infected Mongolian gerbils, detection of the specific amplification started at 5 days PI. This was not possible in other stage of infection (30, 49, 70 and 92 days PI). Our PCR approach in retina demonstrated the presence of the parasite genome before one week PI, while the histological method was not useful for detection of parasite at the retinal and subretinal levels. The PCR in vitreous humor could also be more sensitive than the detection of antibodies by ELISA in the animals in early infection, since prominent positive serology was detectable from 61 days PI. This period in animal challenge, although not strictly equivalent to human infection in clinical course, could be considered as an acute infection in patients regarding the parasitic migration and development. It seems that the transmission of larvae was first from intestine to the liver, lung and eye, and then 5 days PI they migrated to other organs. Sometimes larvae return back to the eye to create lesions at the end of migration. It has been demonstrated that *Toxocara* larvae may migrate and persist in the eye of experimental infected animals. This opinion is challenged by several authors, who argued that *Toxocara* is as a causative ocular toxocariasis [28, 29]. In a relatively similar study, Cho et al., (2007) infected Mongolian gerbils with 1000 *Toxocara* ova and reported that 10% of infective larvae were found before day 5 in liver tissue and also showed that ophthalmoscopically, a motile larva was observed in the retina at 14 days PI [30]. In earlier studies, investigators revealed the parasite DNA by PCR during early infection periods in the animals model for *Trypanosoma cruzi* and *Toxoplasma gondii*, thus permitting the diagnosis of infection earlier than when conventional diagnostic techniques such as microscopy or serology are being used [31, 32].

## 5. Conclusion

Histopathology, serology (ELISA), and molecular (PCR) methods tested for diagnosis of ocular toxocariasis caused by *T. cati* in experimentally infected Mongolian gerbils and Wistar rats revealed that molecular method was the most sensitive and superior compared to both histopathology and ELISA in the early diagnosis of ocular infection. The PCR provided a rapid, reliable and sensitive means by which one establish the exact etiology of such parasitic infection so that optimal therapy can be started promptly. The superior performance of the PCR in detecting infected Mongolian gerbils and Wistar rats in this small experimental population warrants further evaluation in a larger population of animal models and humans naturally infected with *T. cati*. 

## References

[1] Azizi S, Oryan A, Sadjjadi SM, Zibaei M (2007). Histopathologic changes and larval recovery of *Toxocara cati* in experimentally infected chickens. *Parasitology Research*.

[2] Despommier D (2003). Toxocariasis: clinical aspects, epidemiology, medical ecology, and molecular aspects. *Clinical Microbiology Reviews*.

[3] Clemett RS, Williamson HJ, Hidajat RR, Allardyce RA, Stewart AC (1987). Ocular *Toxocara canis* infections: diagnosis by enzyme immunoassay. *Australian and New Zealand Journal of Ophthalmology*.

[4] de Visser L, Rothova A, de Boer JH (2008). Diagnosis of ocular toxocariasis by establishing intraocular antibody production. *American Journal of Ophthalmology*.

[5] Akao N, Ohta N (2007). Toxocariasis in Japan. *Parasitology International*.

[6] Zibaei M, Sadjjadi SM, Uga S (2010). Experimental *Toxocara cati* infection in gerbils and rats. *Korean Journal of Parasitology*.

[7] Dzbenski TH, Hautz W, Bitkowska E (2001). Experimental toxocariasis in rabbits: immunological markers of ocular infections. *Wiadomości Parazytologiczne*.

[8] Sakai R, Kawashima H, Shibui H, Kamata K, Kambara C, Matsuoka H (1998). *Toxocara cati*-induced ocular toxocariasis. *Archives of Ophthalmology*.

[9] Cardillo N, Rosa A, Ribicich M, López C, Sommerfelt I (2009). Experimental infection with *Toxocara cati* in BALB-c mice, migratory behaviour and pathological changes. *Zoonoses and Public Health*.

[10] Taira K, Saitoh Y, Kapel CMO (2011). *Toxocara cati* larvae persist and retain high infectivity in muscles of experimentally infected chickens. *Veterinary Parasitology*.

[11] Zhu XQ, D’Amelio S, Palm HW, Paggi L, George-Nascimento M, Gasser RB (2002). SSCP-based identification of members within the Pseudoterranova decipiens complex (Nematoda: Ascaridoidea: Anisakidae) using genetic markers in the internal transcribed spacers of ribosomal DNA. *Parasitology*.

[12] Borecka A, Gawor J, Niedworok M, Sordyl B (2008). Detection of *Toxocara canis* larvae by PCR in the liver of experimentally infected Mongolian gerbils (*Meriones unguiculatus*). *Helminthologia*.

[13] Akao N, Takayanagi TH, Suzuki R, Tsukidate S, Fujita K (2000). Ocular larva migrans caused by *Toxocara cati* in Mongolian gerbils and a comparison of ophthalmologic findings with those produced by *T. canis*. *Journal of Parasitology*.

[14] Hayashi E, Akao N, Fujita K (2003). Evidence for the involvement of the optic nerve as a migration route for larvae in ocular toxocariasis of Mongolian gerbils. *Journal of Helminthology*.

[15] Lescano SZ, Queiroz ML, Chieffi PP (2004). Larval recovery of *Toxocara canis* in organs and tissues of experimentally infected *Rattus norvegicus*. *Memorias do Instituto Oswaldo Cruz*.

[16] Zibaei M, Sadjjadi SM, Jahadi Hosseini SH, Sarkari B (2007). A method for accelerating the maturation of *Toxocara cati* eggs. *Iranian Journal of Parasitology*.

[17] De Savigny DH (1975). In vitro maintenance of *Toxocara canis* larvae and a simple method for the production of *Toxocara* ES antigen for use in serodiagnostic tests for visceral larva migrans. *Journal of Parasitology*.

[18] Li MW, Lin RQ, Chen HH, Sani RA, Song HQ, Zhu XQ (2007). PCR tools for the verification of the specific identity of ascaridoid nematodes from dogs and cats. *Molecular and Cellular Probes*.

[19] Molk R (1983). Ocular toxocariasis: a review of the literature. *Annals of Ophthalmology*.

[20] Shields JA (1984). Ocular toxocariasis. A review. *Survey of Ophthalmology*.

[21] Gillespie SH, Dinning WJ, Voller A, Crowcroft NS (1993). The spectrum of ocular toxocariasis. *Eye*.

[22] Good B, Holland CV, Taylor MRH, Larragy J, Moriarty P, O’Regan M (2004). Ocular toxocariasis in schoolchildren. *Clinical Infectious Diseases*.

[23] Zanandréa LIDC, Oliveira GM, Abreu AS, Pereira FEL (2008). Ocular lesions in gerbils (*Meriones unguiculatus*) infected with low larval burden of *Toxocara canis*: observations using indirect binocular ophthalmoscopy. *Revista da Sociedade Brasileira de Medicina Tropical*.

[24] Magnaval J-F, Malard L, Morassin B, Fabre R (2002). Immunodiagnosis of ocular toxocariasis using Western-blot for the detection of specific anti-*Toxocara* IgG and CAP*™* for the measurement of specific anti-*Toxocara* IgE. *Journal of Helminthology*.

[25] Bertelmann E, Velhagen K-H, Pleyer U, Hartmann C (2003). Ocular toxocariasis. Diagnostic and therapeutic options. *Ophthalmologe*.

[26] Żarnowska H, Jastrzębska M (1994). Excretory-secretory larval antigens of *Toxocara canis*: physico-chemical characteristics and specificity assayed by Western blot technique. *Acta Parasitologica*.

[27] Sommerfelt IE, Santillán G, Lopez C, Ribicich M, Franco AJ (2001). Immunological and hematological response in experimental *Toxocara canis*-infected pigs. *Veterinary Parasitology*.

[28] Takayanagi TH, Akao N, Suzuki R, Tomoda M, Tsukidate S, Fujita K (1999). New animal model for human ocular toxocariasis: ophthalmoscopic observation. *British Journal of Ophthalmology*.

[29] Santos SV, Lescano SZ, Castro JM, Chieffi PP (2009). Larval recovery of *Toxocara cati* in experimentally infected *Rattus norvegicus* and analysis of the rat as potential reservoir for this ascarid. *Memorias do Instituto Oswaldo Cruz*.

[30] Cho S, Egami M, Ohnuki H (2007). Migration behaviour and pathogenesis of five ascarid nematode species in the Mongolian gerbil *Meriones unguiculatus*. *Journal of Helminthology*.

[31] Weiss LM, Udem SA, Salgo M, Tanowitz HB, Wittner M (1991). Sensitive and specific detection of toxoplasma DNA in an experimental murine model: use of *Toxoplasma gondii*-specific cDNA and the polymerase chain reaction. *Journal of Infectious Diseases*.

[32] Kirchhoff LV, Votava JR, Ochs DE, Moser DR (1996). Comparison of PCR and microscopic methods for detecting *Trypanosoma cruzi*. *Journal of Clinical Microbiology*.

